# An Alternating D1-A-D2-A Conjugated Ternary Copolymer Containing [1,2,5]selenadiazolo[3,4-c]pyridine Unit With Photocurrent Response Up to 1,100 nm

**DOI:** 10.3389/fchem.2020.00255

**Published:** 2020-04-28

**Authors:** Xuelong Huang, Ning Lan, Yunnan Yan, Xin Hu, Shengjian Liu

**Affiliations:** ^1^Department of Pharmaceutical Engineering, College of Pharmacy, Gannan Medical University, Ganzhou, China; ^2^Guangzhou Key Laboratory of Materials for Energy Conversion and Storage, Guangdong Provincial Engineering Technology Research Center for Materials for Energy Conversion and Storage, School of Chemistry, South China Normal University, Guangzhou, China

**Keywords:** D1-A-D2-A, [1,2,5]selenadiazolo[3,4-c]pyridine, polymer photodetectors, near-infrared absorption, ternary copolymer

## Abstract

Two narrow band gap conjugated ternary copolymers comprising two electron-rich (donor, D) and one electron-deficient (acceptor, A) moieties regularly alternating along the polymer backbone were designed and synthesized. The polymers with the repeating unit in a D1-A-D2-A manner were constructed by copolymerizing a bisstannyled-D1 (D1 = n-alkyl-substituted cyclopentadithiophene) and a dibromo-monomer (Br-A-D2-A-Br, D2 = branched-alkyl-substituted cyclopentadithiophene, A =[1,2,5]selenadiazolo[3,4-c]pyridine or 5-fluorobenzo[c][1,2,5]selenadiazole) through a palladium-catalyzed Stille polymerization. This approach that enables variations in the donor fragment substituents can not only control the polymer regiochemistry but also the solubility. Two ternary copolymers exhibited absorbance up to near-infrared region along with relatively narrow band gap in the range of 1.02–1.26 eV. The polymeric photovoltaic cells based on CDTPSE/PC_61_BM show the short circuit density of 1.45 mA cm^−2^, open current voltage of 0.53 V, and photocurrent spectra response from 300 to 1,150 nm under AM 1.5 simulator (100 mW cm^−2^). It is indicated that it can be potentially applied to near infrared photodetectors.

**Graphical Abstract:**
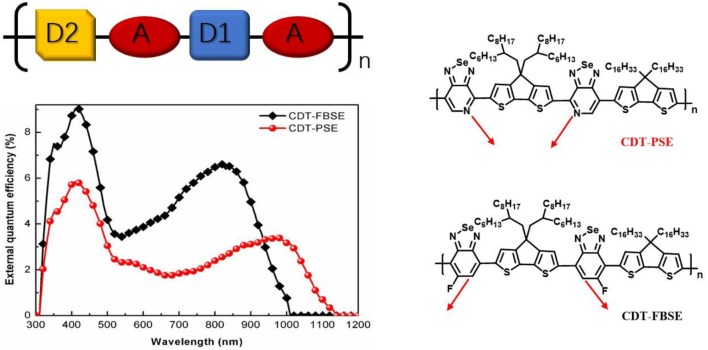
The D1-A-D2-A structure ternary copolymers and the external quantum efficiency.

## Highlights

- Two novel D2-A-D1-A type copolymers containing [1,2,5]selenadiazolo[3,4-c]pyridine or 5-fluorobenzo[c][1,2,5]selenadiazole were synthesized.
- The D2-A-D1-A structure made control over the polymer regiochemistry and solubility possible.
- Broad photocurrent response up to 1,100 nm was realized.

## Introduction

Conjugated polymers have attracted both industrial and academic interests due to the potential for large-area, flexible, and low-cost applications in recent years. Great progress has been made in related research areas based on conjugated polymers including polymer light-emitting diode (PLED), polymeric field-effect transistor (PFET), polymer solar cell (PSC) (Zhao et al., 2016). The conjugated polymers with narrow band gap are promising for the development of polymer photodetectors (PPDs) due to the broad absorption and photoresponse (Gong et al., 2009). Photodetectors from the ultraviolet (UV) to near-infrared (NIR) wavelength is critical for multi-functional applications including chemical/biological sensing, image sensing, environmental monitoring, remote control (Chen et al., 2016). Conventional photodectors exhibited limited absorption ranges through inorganic materials including GaN, Si, InGaAs (Hu et al., 2013). Polymer photodetectors (PPDs) achieve broad spectral response and high sensitivity through photoinduced electron transfer from donor materials (conjugated polymers) to acceptor materials (fullerene derivatives) (Chen and Cao, 2009). For polymer photodetectors applications, donor materials with a narrow band gap are desirable because of their contribution to obtaining photocurrent at long wavelength including NIR region (Hendriks et al., 2014). The common methods to reduce the band gap of conjugated polymers such as extension of conjugation length or introduction of strong donor and acceptor groups can often lead to a rise in the highest occupied molecular orbital (HOMO) energy level (Dong et al., 2013). High-lying highest occupied molecular orbital (HOMO) energy level of conjugated copolymers is essentially unfavorable for ambient stability of devices (Miao et al., 2018). The open circuit voltage (*V*_*oc*_) of OPVs including photodetectors is directly related to the bang gap between the HOMO of donor materials (conjugated polymers) and the lowest unoccupied molecular orbital (LUMO) energies of the acceptor (fullerene). To promote the *V*_*oc*_, the D-A type of conjugated copolymers containing weak donor unit (D, donor) and strong acceptor (A, acceptor) along polymer backbone are synthesized (Han et al., 2017). The “weak donor-strong acceptor” strategy which is widely used in the synthesis of donor materials in OPVs can not only maintain low-lying HOMO energy levels to promote the *V*_*oc*_ but also effectively reduce band gap through intramolecular charge transfer (ICT) (Zhou et al., 2010). It is difficult to balance between selecting weak donor to promote the *V*_*oc*_ value and selecting strong donor to broad spectral range to the NIR. Bazan et al. reported a conjugated polymer containing pyridyl[2,1,3]thiadiazole (PT) acceptor unit with very low *E*_g_-e*V*_*oc*_ loss in OPVs by controlling conjugated polymer regioregularity (Wang et al., 2014). It is possible to fabricate promising PPDs materials with strong donor unit in conjugated polymer by controlling the precise orientation of the donor or acceptor units relative to the backbone vector (Qin et al., 2014).

Compared with pyridyl[2,1,3]thiadiazole (PT), [1,2,5]selenadiazolo[3,4-c]pyridine (PSe) has lower band gaps and the wavelength region has longer in near-infrared (NIR) wavelength than the sulfur based analogs (Hou et al., 2008). In fact, some Se-containing electron-acceptor building blocks, such 2,1,3-benzoselenadiazole and the derivatives are generally poorly soluble. In order to overcome the poor solubility and improve the regularity of the polymer, we proposed regular conjugated ternary copolymers consisting of ternary components with the repeating units denoted as D1–A–D2–A (Huang et al., 2015). This means that the solubility of the D1–A–D2–A structure polymer can be altered by changing of the solubilizing groups on D1 or D2 fragment, respectively. Compared with other well-known donor units, the CDT unit as a strong donor can provide an easy way to incorporate solubilizing substituents (Zhou et al., 2012). For the acceptor fragment, [1,2,5]selenadiazolo[3,4-c]pyridine (PSe) and 5-fluorobenzo[c][1,2,5]selenadiazole (FBSe) are selected due to the similar property to the S-containing analog, which demonstrates asymmetric reactivity between ortho- and meta-positions (Sun et al., 2012).

## Results and Discussion

### Synthesis and Characterization

**Scheme 1:**
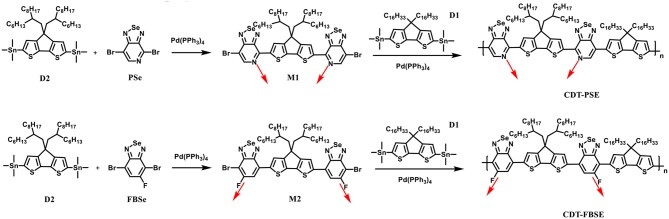
Synthetic route of D1-A-D2-A ternary copolymers.

A stepwise synthetic strategy is required for the preparation of the D1-A-D2-A polymers, see Scheme 1. First, the palladium catalyzed Stille coupling reaction of two equivalents of 4,7-dibromo-[1,2,5]selenadiazolo[3,4-c]pyridine with (4,4-bis(2-hexyldecyl)-4H-cyclopenta[2,1-b:3,4-b′]dithiophene-2,6-diyl)bis(trimethylstannane) (D2) provided 4,4′-(4,4-bis(2-hexyldecyl)-4H-cyclopenta[2,1-b:3,4-b′]dithiophene-2,6-diyl)bis(7-bromo-[1,2,5]selenadiazolo[3,4-c]pyridine) (M1). The palladium catalyzed Stille coupling reaction of the D2 with 2 equivalents of 4,7-dibromo-5-fluorobenzo[c][1,2,5]selenadiazole (FBSe) gave the dibromointermediate of 7,7′-(4,4-bis(2-hexyldecyl)-4H-cyclopenta[2,1-b:3,4-b′]dithiophene-2,6-diyl)bis(4-bromo-5-fluorobenzo[c][1,2,5]selenadiazole), which was denoted as M2 (Supplementary Figures 1–7).

The asymmetric nature of PSe allows for monofunctionalization resulting in strictly organized PSe orientations, such that the pyridyl nitrogen atoms point toward the CDT fragment in M1 intermediate. For another acceptor fragment, FBSe has the asymmetric reactivity in meta-position, in which the fluorine atoms are far from the CDT fragment in M2 intermediate. M1 and M2 are subsequently polymerized with (4,4-dihexadecyl-4H-cyclopenta[2,1-b:3,4-b′]dithiophene-2,6-diyl)bis(trimethylstannane) (D1) under microwave heating in anhydrous toluene to yield two regioregular, D1-A-D2-A polymers CDT-PSE and CDT-FBSE.

The two polymers were purified by Soxhlet extraction by using methanol, acetone, and hexane successively to remove the oligomer and catalyst. The number-average molecular weight (*M*_n_) and polydispersity index (PDI) of the two polymers were determined by gel permeation chromatography (GPC) at 150°C using 1,2,4-trichlorobenzene as the eluent and polystyrene standards. The number-average molecular weight (*M*_n_) was determined to be 21.9 and 46.1 kDa for CDT-PSE, CDT-FBSE, respectively, with PDI values of 2.7 and 2.3, respectively. Thermal properties of the two polymers were detected by differential scanning calorimetry (DSC) and thermogravimetric analysis (TGA) measurements. No discernable phase transition was realized in DSC characteristics up to 300°C. TGA measurements demonstrated that the decomposition temperatures (corresponding to 5% weight-loss temperatures) were 354.3 and 365.2°C for CDT-PSE and CDTF-BSE, respectively, implying good thermal stabilities of the two polymers (Supplementary Figure 8).

### Optical Properties

**Figure 1:**
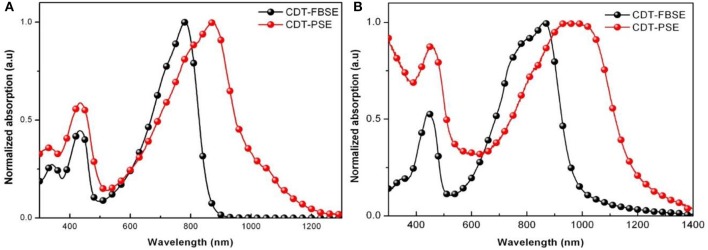
Normalized UV-vis absorption spectra of D2-A-D1-A polymers in *o*-DCB solution **(A)** and in thin films **(B)**.

Absorption spectra in the 300–1,400 nm region of CDT-PSE and CDT-FBSE in *o*-dichlorobenzene solution with concentration of ca. 1 × 10^−5^ g mL^−1^ as well as in thin films are shown in Figure 1. Two polymers show two absorption bands. The high energy absorbance band located in the range of 300–500 nm can be attributed to the π-π^*^ transition of D1–A–D2–A backbones in dilute solutions, while the low energy absorbance peak displayed typical dual band features of D–A copolymers with the intramolecular charge transfer (ICT) effects between the donors and the acceptor moieties located at long wavelength up to NIR regions. Broader and further red-shifted bands arise in the solid-state absorption spectra. The maximum absorption edge of CDT-PSE and CDT-FBSE was at 1,200 and 1,000 nm, respectively. Relative to CDT-FBSE, the absorption peak of CDT-PSE as a thin film was broader in width, which is consistent with the greater degree of structural order due to the PSe nitrogen atoms. The optical band gaps (*E*opt g) of the two D1–A–D2–A polymers as estimated from the onset of the absorbance of solid films are in the range of 1.02–1.26 eV.

### Electrochemical Properties

**Figure 2:**
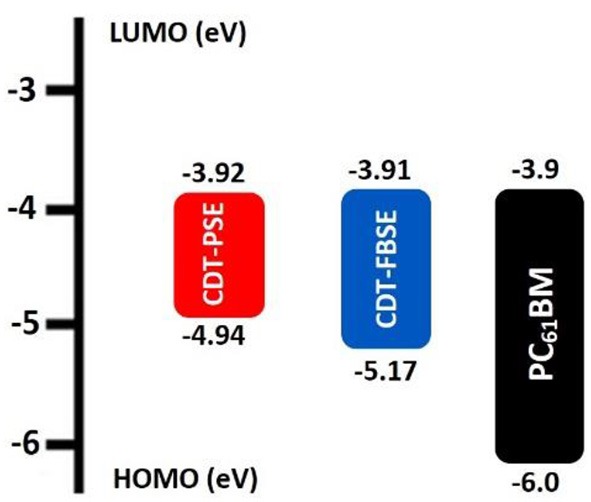
Schematic energy level diagrams of ternary copolymers.

To investigate the influence of regioregularity on the frontier energy levels of the D1–A–D2–A structure polymer, cyclic voltammetry (CV) measurements were performed. The measurement was carried out in tetra(*n*-butyl)ammonium hexafluorophosphate (*n*-Bu_4_NPF_6_, 0.1 M in acetonitrile) solution by using a ITO glass electrode as working electrode and saturated calomel electrode (SCE) as a reference electrode at a scan rate of 50 mV s^−1^. The films of CDT-PSE and CDT-FBSE were cast from *o*-DCB solution with a concentration of 10 mg mL^−1^ and all measurements were performed under an inert atmosphere. The CV characteristics of two polymers are demonstrated in Figure 2. It is assumed that the redox potential of Fc/Fc+ was measured as a standard, which has an absolute energy level of −4.80 eV to vacuum. The onset of oxidation (*E*_ox_) of the two polymers was estimated to be 0.14, and 0.37 eV for CDT-PSE and CDT-FBSE in the same experimental conditions, respectively (Supplementary Figure 9).

Therefore, the highest occupied molecular orbitals (*E*_HOMO_) of the two polymers were calculated according to the following equation of *E*_HOMO_ = –e(*E*_ox_+4.80) (eV). Consequently, the HOMO values are −4.94 and −5.17 eV for CDT-PSE and CDT-FBSE, respectively. According to the equation of *E*_LUMO_ =-*E*_HOMO_ + *E*opt g, the *E*_LUMO_ are calculated to be −3.92 and −3.91 eV for CDT-PSE and CDT-FBSE, respectively. Detailed electrochemical data are summarized in Table 1 and Figure 2.

**Table 1:** Electrochemical properties and molecular weight of the polymers.

	***E*_HOMO_^a^(ev)**	***E*_LUMO_^a^(ev)**	**$E_{g}^{opt\;b}$(ev)**	***M*_**n**_(kDa)**	**PDI**	**T_**d**_ (^**°**^C)**
CDT-PSE	−4.94	−3.92	1.02	21.9	2.7	354.3
CDT-FBSE	−5.17	−3.91	1.26	46.1	2.3	365.2

a *The E_HOMO_ is determined by cyclic voltammetry, E_LUMO_ = -E_HOMO_ + $E_{g}^{opt}$*.

b *Optical band-gap estimated from the absorption onset as thin films*.

### Photovoltaic Properties

**Figure 3:**
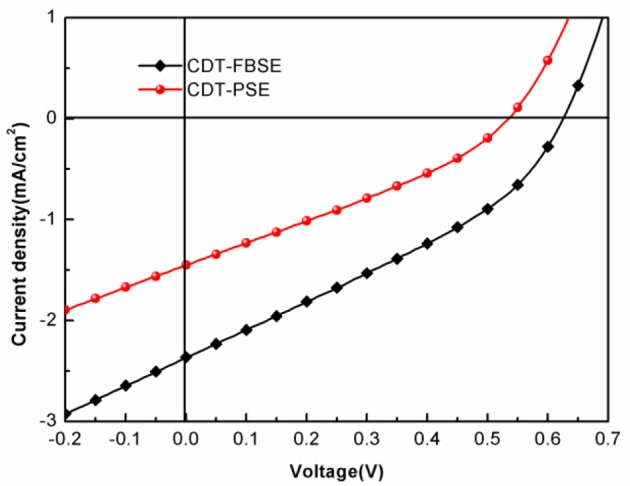
*J-V* characteristics of the devices with the structure of ITO/PFN-OX/polymer: PC_61_BM (1: 1, wt:wt)/MoO_3_/Al under AM 1.5G irradiation (100 mW cm^−2^).

**Figure 4:**
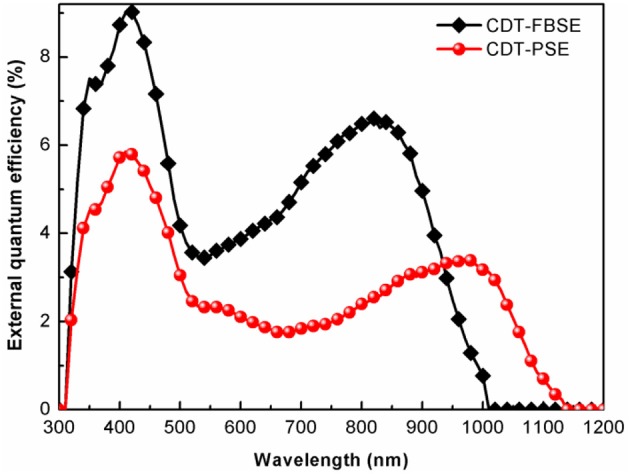
EQE of the photovoltaic devices with configuration of ITO/PFN-OX/polymer: PC_61_BM(1: 1, wt:wt)/MoO_3_/Al.

The photovoltaic properties of the two polymers were investigated by fabricating solar cells with an inverted device structure of ITO/poly[9,9-bis(60-(*N, N*-diethylamino)hexyl)fluorene-alt-9,9-bis(3-ethyl(oxetane-3-ethyloxy)hexyl)fluorene] (PFN-OX)/polymer:PC_61_BM/MoO_3_/Al. The PFN layer (~5 nm) was deposited on the top of photoactive layer before deposition of cathode, since it can facilitate electrons extraction from photoactive layer and reduce the work function of ITO. The thickness of MoO_3_ layer is about 10 nm and Al electrode thickness is about 80 nm. The photoactive layer of the polymer: PC_61_BM was fabricated by spin coating from *o*-dichlorobenzene (*o*-DCB) solution. The current density vs. voltage (*J*–*V*) characteristics of the solar cell devices under AM 1.5 G at 100 mW m^−2^ conditions was shown in Figure 3. CDT-PSE was achieved a power conversion efficiency (PCE) of 0.24% with an open circuit voltage (*V*_*oc*_) of 0.53 V, a short-circuit current density (*J*_*sc*_) of 1.45 mA cm^−2^, and a fill factor (FF) of 0.30. The better device performance was achieved based on CDT-FBSE: PC_61_BM as the photoactive layer, which showed a moderate PCE = 0.49% (*V*_*oc*_ =0.63 V, *J*_*sc*_ = 2.37 mA cm^−2^, and FF = 33.2%). We speculated that the moderate value in *V*_*oc*_ can be attributed to the decreased HOMO level. The decreased short circuit current (*J*_*sc*_) in CDT-PSE can be attributed to the lack of driving force for the charge separation in the interface of polymer and PC_61_BM due to the relatively low-lying LUMO energy level of CDT-PSE. The external quantum efficiency (EQE) curves are shown in Figure 4. Both the polymers show a broad EQE response from 300 nm to NIR region. The photosensitivity response of CDT-FBSE is higher over a wide range compared to CDT-PSE. But it is worth noting that CDT-PSE exhibits prolonged response beyond 1,100 nm, which reveals its great potential for the application in NIR photodetector.

### Morphology

**Figure 5:**
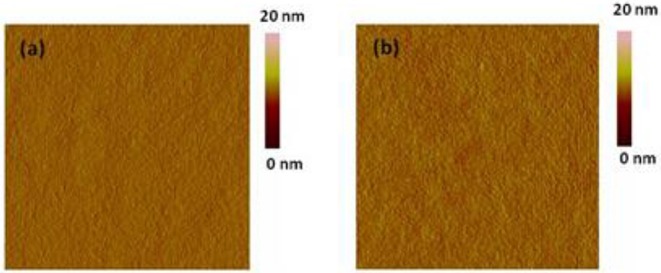
AFM height images of polymer: PC_61_BM blend films (1:1, wt:wt): CDT-PSE **(A)**, CDT-FBSE **(B)**. All the images are in the size of 5 × 5 μm.

Tapping mode atomic force microscopy (AFM) was investigated the surface topography of the bulk heterojunction polymer: PC_61_BM (1:1 in wt:wt) blend films (Figure 5). The blend film of CDT-PSE: PC_61_BM showed comparatively smooth surface topography with a root-mean-square roughness (RMS) value of 0.47 nm. From Figure 5B one can clearly observe the rough morphology of CDT-FBSE: PC_61_BM with a root-mean-square (RMS) roughness of 0.42 nm. The smooth surface topography of the polymer/PC_61_BM components may give rise to this non-optimal nanostructure, which is essentially not favorable for charge carrier transporting to the corresponding electrodes and would lead to relatively low *J*_*sc*_ of the device.

## Conclusion

In conclusion, two novel D1-A-D2-A ternary copolymers containing asymmetric reactivity unit ([1,2,5]selenadiazolo[3,4-c]pyridine or 5-fluorobenzo[c][1,2,5]selenadiazole) was synthesized by stepwise approach. Incorporating the branched-alkyl-substituted cyclopentadithiophene (D2) to the A-D-A intermediates is of great benefit to solubility and purification of the PSe or FBSe units. We have shown that the D1-A-D2-A repeating structure can control the regiochemistry of the polymer backbone and indicate a much lower *E*_g_-e*V*_*oc*_ loss than the D-A analogs. We have presented the n-alkyl-substituted cyclopentadithiophene (D1) to enhance the intermolecular π-stacking interactions. The polymers CDT-PSE and CDT-FBSE show wide light absorption ranges of 300–1,000 and 300–1,100 nm in solid thin film, respectively. The photoresponse wavelengths of the CDT-PSE based on CDT-PSE/PC_61_BM blends extend to about 1,100 nm, illustrating its great potential for the application in NIR photodetector.

## Experimental Section

### Materials

All reagents were purchased from commercial sources such as from Aldrich, Acros and TCI Chemical Co. The ^1^H and ^13^C NMR spectra were measured on a Bruker AV-300 (300 MHz) in a deuterated chloroform solution with tetramethylsilane(TMS) as the internal standard. Compound 4H-cyclopenta[2,1-b:3,4-b′]dithiophene (2), 4,4-dihexadecyl-4H-cyclopenta[2,1-b:3,4-b′]dithiophene (3),(4,4-dihexadecyl-4H-cyclopenta[2,1-b:3,4-b′]dithiophene-2,6-diyl)bis(trimethylstannane) (D1), (4,4-bis(2-hexyldecyl)-4H-cyclopenta[2,1-b:3,4-b′]dithiophene-2,6-diyl)bis(trimethylstannane) (D2), 5-fluorobenzo[c][1,2,5]thiadiazole(5), 4,7-dibromo-5-fluorobenzo[c][1,2,5]thiadiazole (6) were synthesized according to the reported procedures.

### Synthesis of Intermediates

#### Compound 3,6-dibromo-4-fluorocyclohexa-3,5-diene-1,2-diamine (7)

**Figure 6:**
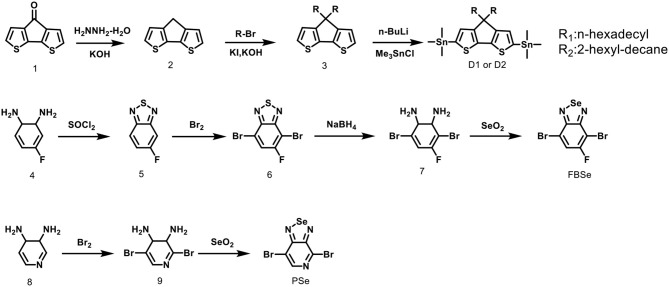
Synthetic routes of monomers.

Compound 7 was synthesized through the reduction of compound 6 with sodium borohydride in ethanol (Figure 6). Compound 6 (5 g, 16 mmol) and ethanol (100 mL) were added to a two-necked round-bottom flask and cooled to 0°C. After sodium borohydride (12.1 g, 320 mmol) was slowly added, the reaction mixture was stirred for 20 h at room temperature. The ethanol was evaporated and the extract was concentrated to obtain pale-yellow compound 7 in 90% yield. ^1^H NMR (300 MHz, CDCl_3_, TMS) (ppm): 6.35 (s, 1H); 5.11 (dd, 4H).

#### Compound 4,7-dibromo-5-fluorobenzo[c][1,2,5]selenadiazole (FBSe)

Compound 7 (0.76 g, 2.67 mmol) and ethanol (60 mL) were added to a two-necked round-bottom flask and heated to refluxing with stirring. After a solution of selenium dioxide (0.312 g, 2.8 mmol) in hot water (5 mL) was slowly added, the reaction mixture was refluxed for 3 h and cooled to room temperature. Then, the precipitate was filtrated, recrystallized from ethyl acetate, and dried. Compound FBSe was obtained as a golden-yellow needle crystal in 87% yield. ^1^H NMR (300 MHz, CDCl_3_, TMS) (ppm): 7.85 (d, 1H).

#### Compound 2,5-dibromo-3,4-dihydropyridine-3,4-diamine (9)

Compound 9 was synthesized through the reaction of compound 8 (9.8 g, 90 mmol) and bromine in hydrobromic acid (48%). The bromine (15 mL) was added dropwise to the vigorously stirred solution at room temperature and the mixture was refluxed for 5 h. Finally, the reaction mixture was cooled to room temperature and filtrated, washed with saturated aqueous sodium thiosulfate solution and distilled water, recrystallized from methanol. Finally, compound 9, the yellow needle crystal was obtained in 51% yield. ^1^H NMR (300 MHz, CDCl_3_, TMS) (ppm): 7.52 (s, 1H); 5.97 (d, 2H); 5,03 (d, 2H).

#### Compound 4,7-dibromo-[1,2,5]selenadiazolo[3,4-c]pyridine (PSe)

Compound 7 (8 g, 30 mmol) and ethanol (120 mL) were added to a two-necked round-bottom flask and heated to refluxing with stirring. After a solution of selenium dioxide (3.34 g, 30 mmol) in hot water (5 mL) was slowly added, the reaction mixture was refluxed for 3 h and cooled to room temperature. Then, the precipitate was filtrated, recrystallized from ethyl acetate, and dried. Compound PSe was obtained as a golden-yellow needle crystal in 90% yield. ^1^H NMR (300 MHz, CDCl_3_, TMS) (ppm): 8.74 (s, 1H).

### Measurement and Characterization

^1^H and ^13^C NMR spectra were measured on Bruker AVANCE Digital 300 MHz NMR workstation or Bruker AVANCE Digital 400 MHz NMR workstation. The number-average molecular weights (*M*n) were determined by Polymer Laboratories PL220 Chromatograph (150°C in 1,2,4-trichlorobenzene) with linear polystyrene as the standard. Cyclic voltammograms (CV) were recorded on CHI 600D electrochemical workstation at a scan rate of 50 mV s^−1^. The tapping-mode atomic force microscopy images were performed on a Nano-Scope NS3A system (Digital Instrument) to observe the surface morphologies of the ITO-coated glass substrates. UV-vis spectra were obtained by a HP 8453 spectrophotometer. Thermogravimetric analyses (TGA) measurements were carried out with a NETZSCH TG 209 under a heating rate of 10°C min^−1^ and a N_2_ flow rate of 20 mL min^−1^. Differential scanning calorimetry (DSC) were conducted on a Netzsch DSC 204 under N_2_ flow at heating and cooling rates of 10°C min^−1^.

## Solar Cell Device Fabrication and Characterization

PSCs were fabricated with an inverted device structure of ITO/PFN-OX/polymer:PC_61_BM/MoO_3_/Al. ITO-coated glass substrates were cleaned in ultrasonic baths by sequentially immersing the substrates in detergent, deionized water, acetone and isopropyl alcohol and dried under a nitrogen stream, followed by a UV-ozone treatment. A 5 nm thin film of polymer PFN-OX (0.5 mg mL^−1^) was spin-casted on the pre-cleaned ITO substrates, and the PFN-OX films were thermally annealed at 150°C for 20 min. The polymer: PC_61_BM active blend layer with a thickness of ~90 nm was prepared by spin-coating the 1,2-dichlorobenzene solution on top of the PFN-OX layer. Finally, 10 nm molybdenum oxide (MoO_3_) and 100 nm aluminum were deposited by thermal evaporation under vacuum (~10^−6^ mbar) in glovebox to complete device fabrication. The characteristic current-voltage (*J*–*V*) curves of the resulting PSCs were measured using a Keithley 236 source meter under 100 mW cm^−2^ (1 sun, AM 1.5G spectra, calibrated using a standard Si solar cell and simulator provided by Oriel model 91192). The external quantum efficiency (EQE) measurements were performed by using a QE-R3011 system. A calibrated Si photodiode was used to determine the photosensitivity.

## Data Availability Statement

The raw data supporting the conclusions of this article will be made available by the authors, without undue reservation, to any qualified researcher.

## Author Contributions

XHua: investigation, data curation, and writing-original draft. NL: writing-review & editing. YY: investigation. XHu: data curation. SL: project administration.

## Conflict of Interest

The authors declare that the research was conducted in the absence of any commercial or financial relationships that could be construed as a potential conflict of interest.
