# Supplementary material for: An Alternating D1-A-D2-A Conjugated Ternary Copolymer Containing [1,2,5]selenadiazolo[3,4-c]pyridine Unit With Photocurrent Response Up to 1,100 nm
Source: Front Chem. 2020 Apr 28;8:255. doi: 10.3389/fchem.2020.00255 (PMC7198836; doi:10.3389/fchem.2020.00255)
Supplement: Supplementary file 1 [file Data_Sheet_1.PDF]

Supplementary Material

An alternating D1-A-D2-A conjugated copolymer containing [1,2,5]selenadiazolo[3,4-c]pyridine unit with photocurrent response up to 1100nm

Table of Contents

1. **Figure S1-S7**. **NMR** Spectrum of monomers in $CDCl_3$**……………………………………………**S2-S5

2. **Figure S8**. Thermal gravimetric characteristics of polymers**……………………………………**S5

3. **Figure S9**. Cyclic voltammograms of copolymers**………. …………….……….…….….…….**S6

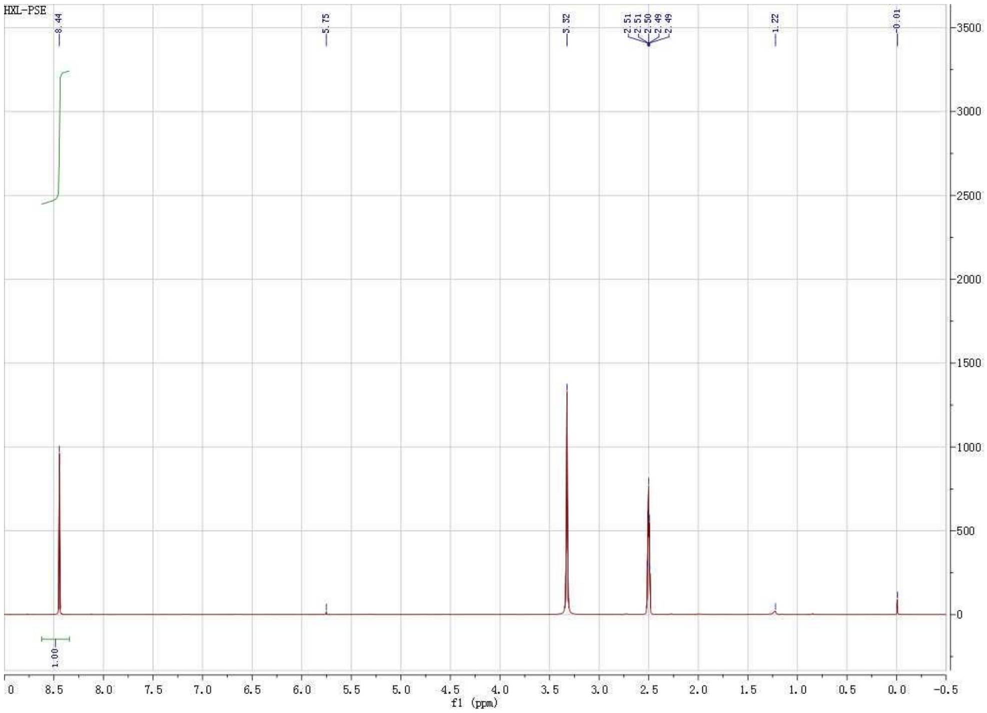

Supplementary Figure 1. ^{1}H NMR of Pse

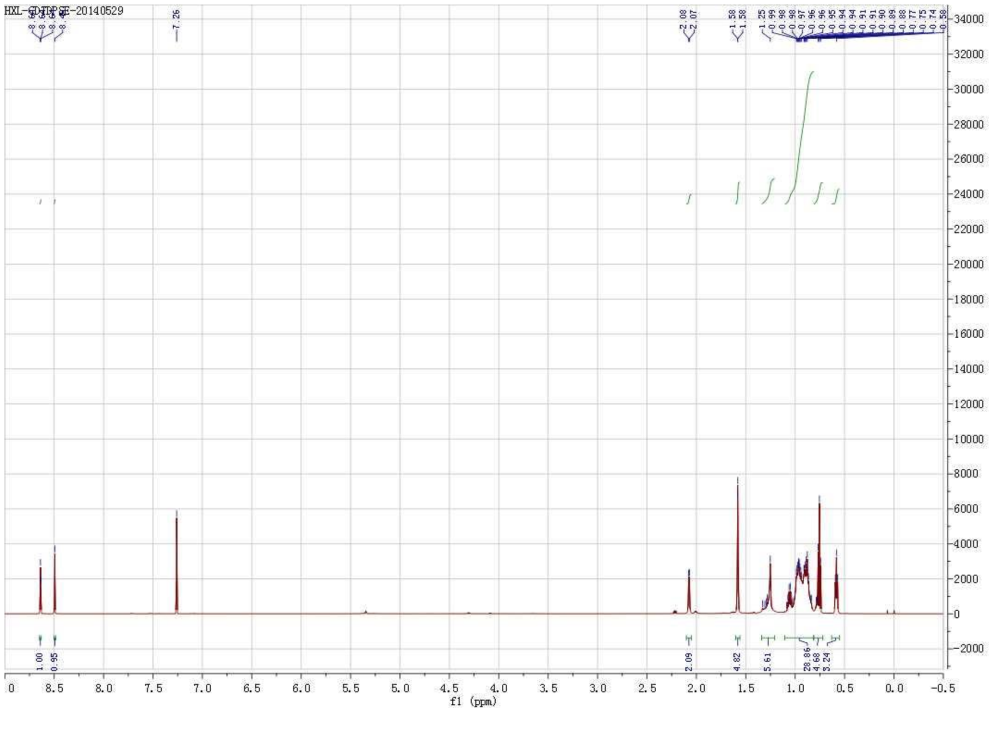

Supplementary Figure 2. ^{1}H NMR of M1

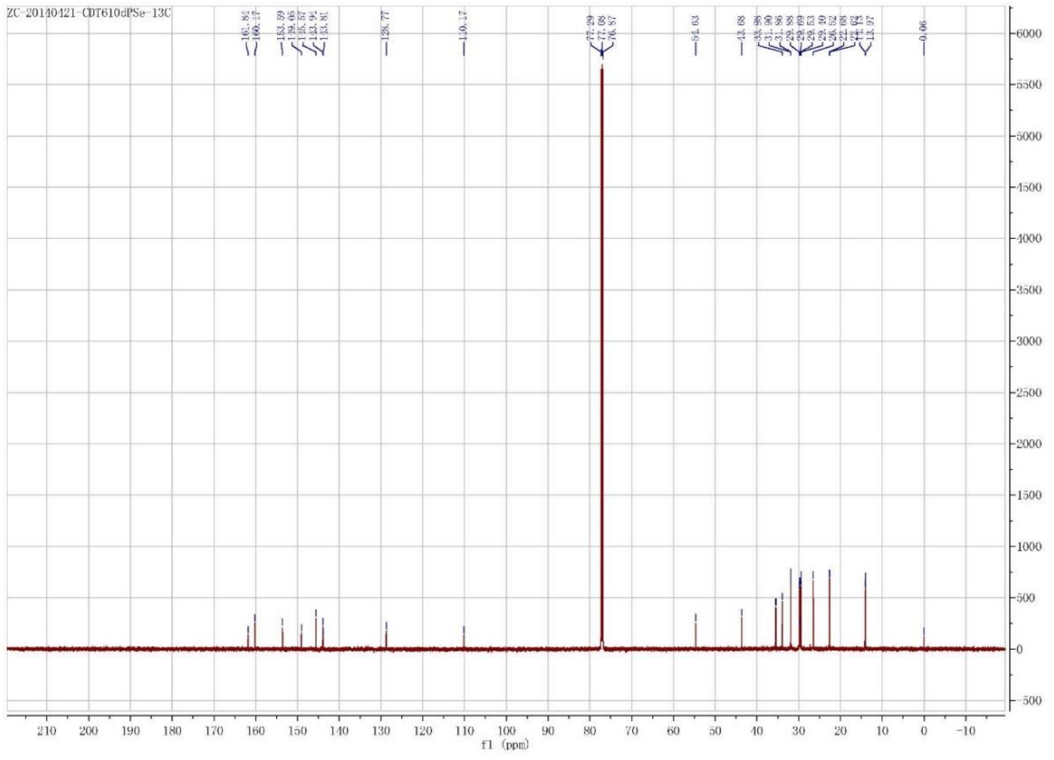

Supplementary Figure 3. ^{13}C NMR of M1

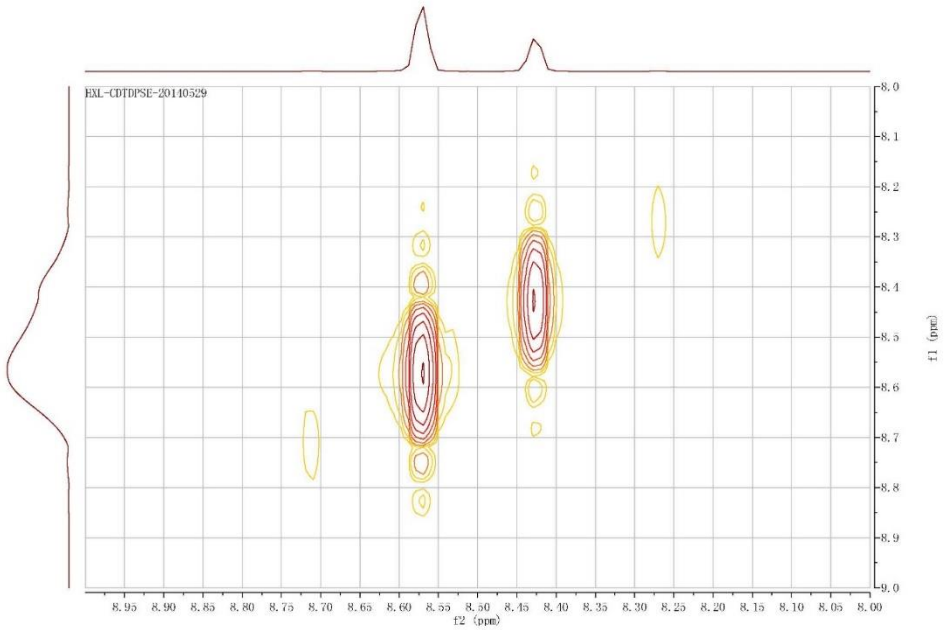

Supplementary Figure 4. ^{1}H NMR and NOESY spectra of M1

S3

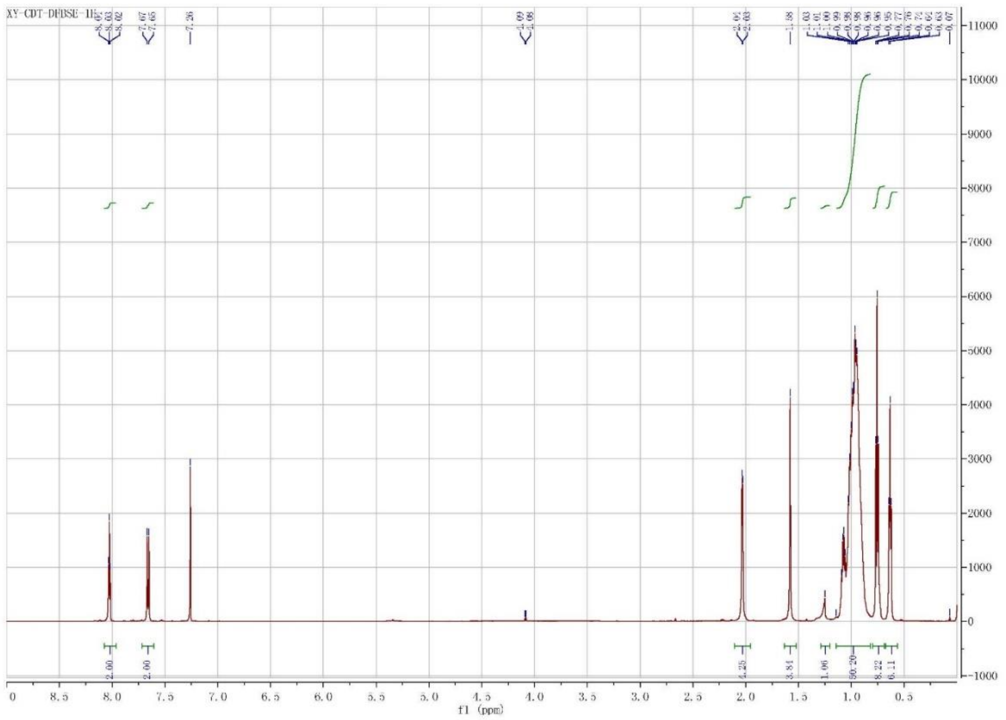

Supplementary Figure 5. ^{1}H NMR of M2

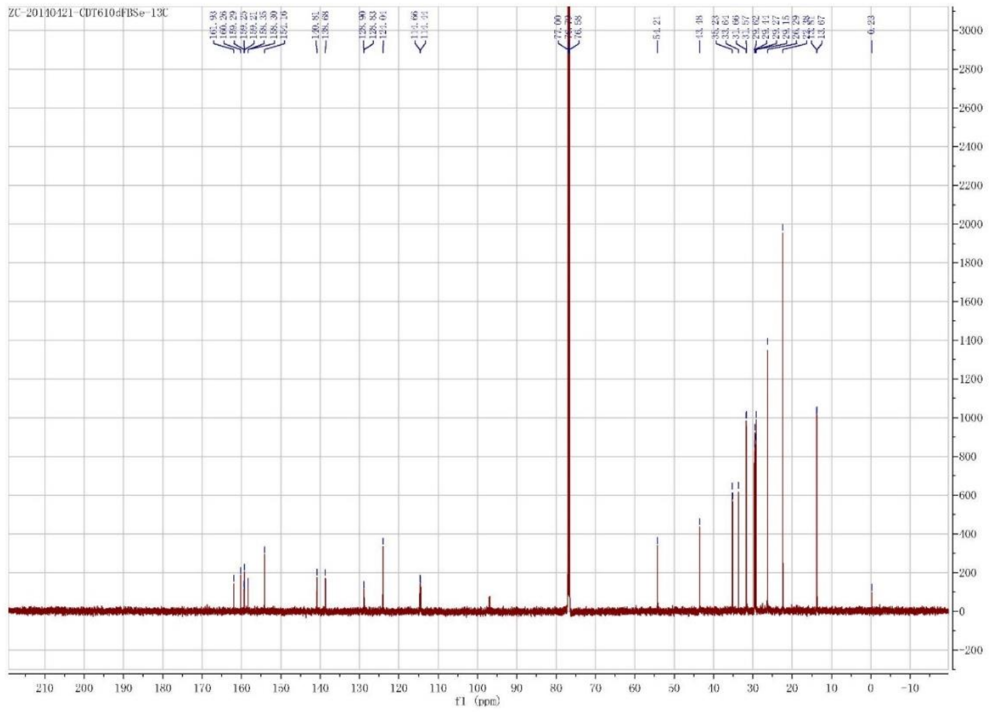

Supplementary Figure 6. ^{13}C NMR of M2

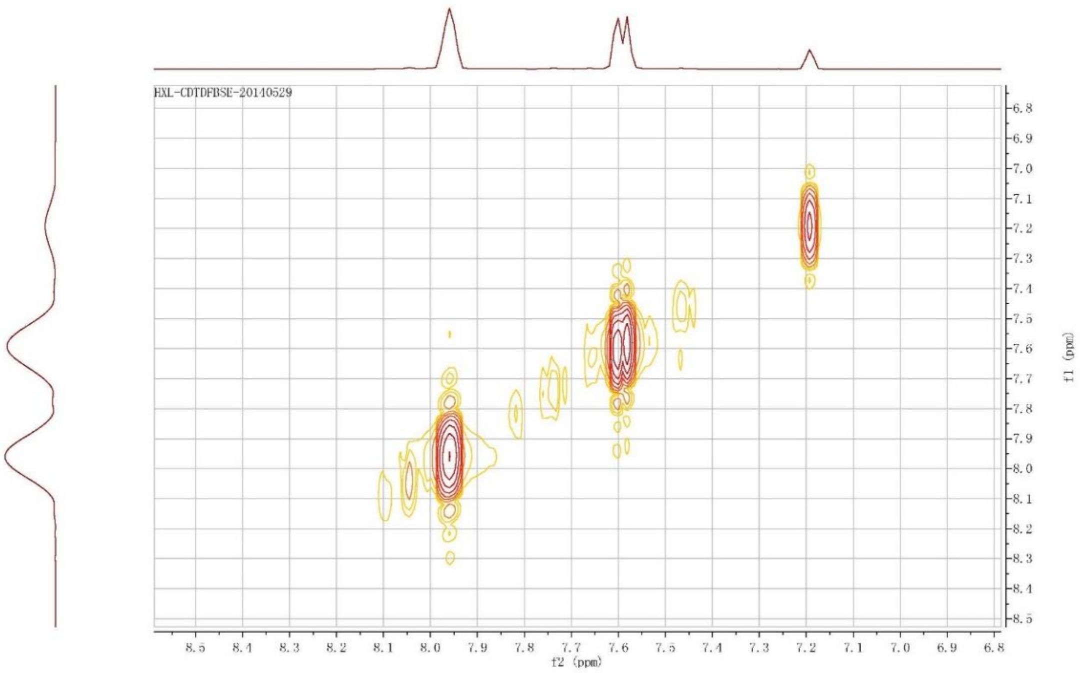

Supplementary Figure 7. ^{1}H NMR and NOESY spectra of M2

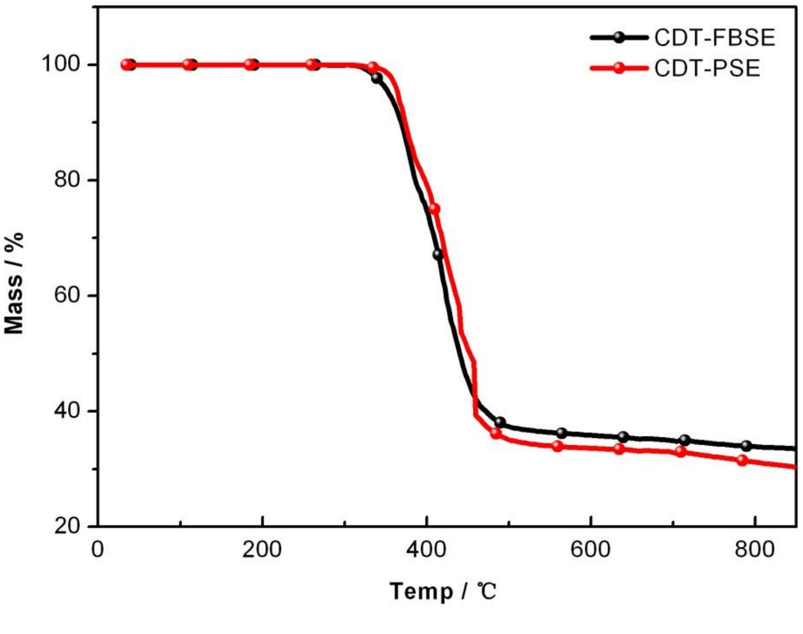

Supplementary Figure 8. Thermal gravimetric characteristics of polymers

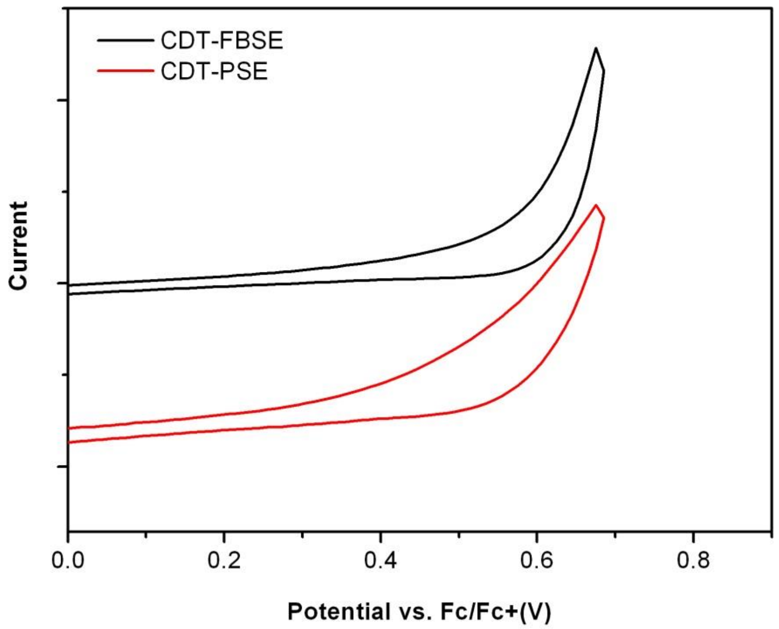

Supplementary Figure 9. Cyclic voltammograms of polymers
